# Autonomous platforms for data-driven organic synthesis

**DOI:** 10.1038/s41467-022-28736-4

**Published:** 2022-02-28

**Authors:** Wenhao Gao, Priyanka Raghavan, Connor W. Coley

**Affiliations:** 1 Department of Chemical Engineering, Massachusetts Institute of Technology, Cambridge, MA 02139 USA; 2 Department of Electrical Engineering and Computer Science, Massachusetts Institute of Technology, Cambridge, MA 02139 USA

**Keywords:** Automation, Chemical engineering, Cheminformatics, Organic chemistry

## Abstract

Achieving autonomous multi-step synthesis of novel molecular structures in chemical discovery processes is a goal shared by many researchers. In this Comment, we discuss key considerations of what an ideal platform may look like and the apparent state of the art. While most hardware challenges can be overcome with clever engineering, other challenges will require advances in both algorithms and data curation.

## A framework for autonomous synthesis

In the iterative design, synthesis, and testing of new functional molecules, the rate at which candidate molecules can be physically realized often limits the rate at which computational designs can be validated. Platforms capable of performing chemical reactions in an automated or semi-automated manner, where the physical operations of a chemist are replaced by robotics and the planning by data-driven algorithms, can potentially mitigate this bottleneck.

**Fig. 1:**
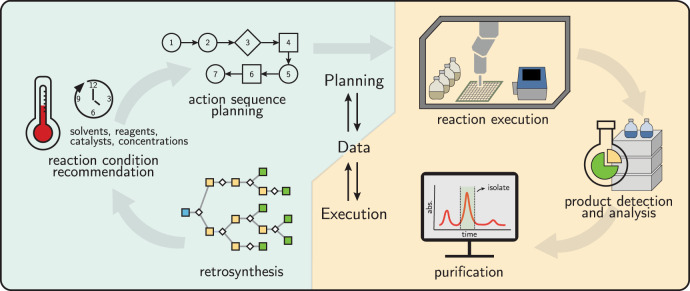
A high-level workflow for autonomous data-driven organic synthesis. This process requires the close integration of synthesis planning and synthesis execution, linked by data that captures details of reaction and purification processes and their outcomes.

The actualization of autonomous, data-driven organic synthesis will rely on advances in both *hardware* and *software* capabilities to overcome a combination of both *practical* and *scientific* challenges^1^. In this Comment, we outline the major considerations that must be made to design autonomous platforms for target-oriented synthesis, progressing from the execution hardware, to synthesis planning, to adaptiveness and error handling, and finally to self-learning (Fig. 1).

## Hardware requirements and desiderata

The basis of automated chemistry is the modularization of common physical operations to perform reactions: transferring a prescribed amount of starting materials to a reaction vessel, heating or cooling that vessel while mixing, purifying/isolating the desired product, analyzing the product, and using it in subsequent reaction steps. Fortunately, many requisite hardware units for such tasks have already been commercialized, such as liquid handling robots, robotic grippers for plate or vial transfer, computer-controlled heater/shaker blocks, and autosamplers for analytical instrumentation. Therefore, a straightforward (but not simple) paradigm for automated chemistry is to automate operations and sample transfer steps between existing lab hardware, exemplified by Burger et al.’s mobile robot chemist^2^.

At the core of organic synthesis, automated reactions are run either in a flow or batch manner, with stirring, heating, and/or cooling capabilities. Key additional considerations when designing these automation components include minimizing evaporative losses, performing air-sensitive chemistries, and maintaining precise temperature control; all are addressable through engineering. An early platform, ChemKonzert, automated multi-step syntheses by replacing manual transfer operations with pumps but otherwise adhering to a typical batch process in round bottom flasks, separation flasks, and filters^3^—a paradigm since advanced and expanded upon by the Chemputer^4^. Additionally, from a practical standpoint, accessing a diverse chemical space requires equipping the platform with a suitably large chemical inventory of building blocks and reagents, otherwise these must be manually prepared prior to any synthesis. In medicinal chemistry applications, Eli Lilly has been a leader in automated multi-step synthesis by designing a platform around microwave vials as reaction vessels^5^, with significant ongoing investments in automation more broadly including a chemical inventory able to store five million compounds^6^. Flow platforms too can be automated for target-oriented synthesis using computer-controlled pumps and flowpath reconfiguration^7,8^, though they require additional planning considerations (e.g., solubility).

Following the reaction, liquid chromatography–mass spectrometry (LC/MS) is most commonly used for analysis or quantitation. Multi-step reactions add a layer of complexity, as crude products must be isolated and resuspended in solvent between reactions. This invites new challenges with regards to the automation of solution transfer between the reaction area and the purification and analysis unit. Constraining the reaction space to a specific subset can mitigate the burden of purification as exemplified by Burke’s iterative MIDA-boronate coupling platform that uses a catch and release method applicable to a specific reaction;^9^ however, a universally applicable purification strategy does not yet exist.

## Synthetic planning including and beyond retrosynthesis

At a high-level, an autonomous chemistry platform must decide what it *intends* to do (as a chemist might describe it) and then translate that into what it should *actually* do (in terms of physical operations). The latter step is dependent on available hardware operations, but can leverage protocols intended to be hardware-agnostic, such as the chemical description language (XDL)^10^. When targeting structures without known experimental procedures, the former step requires developing software tools for computer-aided synthesis planning, including and beyond retrosynthesis.

Computer-aided retrosynthesis has arguably existed almost since the concept of retrosynthesis itself, but has failed to gain traction due to a perception that proposed routes are of low quality. As a turning point, Segler et al.^11^ pioneered a data-driven approach using a Monte Carlo tree search that passed a “chemical Turing test”, wherein graduate-level organic chemists expressed no statistically significant preference between literature-reported routes and the program’s. Mikulak-Klucznik et al.^12^ further demonstrated that this approach is viable for complex natural products with their expert (not data-driven) program, Synthia. Their successes have catalyzed an interest in developing neural models that learn allowable chemical transformations from reaction databases. These models are commonly divided into template-based and template-free approaches depending on whether the model makes use of symbolic pattern-matching rules. Both types have been incorporated with semi-automated synthesis platforms to streamline target-oriented organic synthesis, for example, with Coley et al.’s ASKCOS^7^ and IBM’s integration with commercial hardware^13^. However, successful applications of data-driven retrosynthesis with automation have been relatively simple molecules, where few (1–5) steps are required and where stereocenters are typically sourced from building blocks rather than installed.

It is essential to recognize that retrosynthesis is merely the first step of autonomous organic synthesis, as it does not address numerous practical considerations. Experimental execution of a synthetic route requires specification of quantitative reaction conditions—amounts of each reactant, solvent(s), temperature, time, etc.—and how this translates into a detailed action sequence for the hardware to follow, at the very least specifying order of addition. Subtle changes in procedure can significantly affect the reaction outcomes, but these subtleties are missing from current databases, and therefore are also missing from current data-driven tools^14^. Proposed synthetic routes also require further scoring and ranking in terms of their automation compatibility, perhaps tailored to a specific hardware platform, not just chemical feasibility. These steps remain largely unaddressed.

## Error handling and robustness to mispredictions

The need for precise planning can be partially mitigated with platforms that can cope with mispredictions and are able to adaptively determine a suitable action sequence through trial and error. The optimization of reaction yields or selectivities by modulating reaction conditions is a decades-old task, with recent demonstrations including applications of statistical optimization to multi-step flow chemistry^15^, as well as applications of Bayesian optimization^16^. Predicted conditions may be suitable as an initial guess to be further improved through empirical optimization. This workflow of incorporating reaction screening and optimization between synthesis planning and multi-step synthesis is exemplified by SRI’s SynFini^8^.

However, even this basic level of adaptivity relies on a number of capabilities that are not trivial to automate, such as confirming product identities and quantifying their yields without relying on a user-provided product standard or calibration curve. Most platforms are currently equipped with only LC/MS, while structural elucidation or quantitation may require instruments such as nuclear magnetic resonance (NMR) or corona aerosol detection (CAD), the latter of which promises to enable universal calibration curves. While the hardware challenges can be overcome through engineering efforts, the computer-assisted structural elucidation and prediction of analytical response factors would benefit from renewed attention.

“Failures” in autonomous target-oriented synthesis will often be more dire than a subpar yield. Chemistry is sufficiently complex that predictive models might never be perfectly predictive of physical reality; moreover, we may wish to explore *new* reactivity, which by some definitions is inherently less predictable. A key reaction step might not produce any desired product, warranting a complete revision of that route to circumvent that false-positive prediction. Flow chemistry platforms may be prone to clogging and therefore necessitate a means to detect and recover from such events. Plate-based or vial-based platforms are in principle more robust, provided that the reaction vessel is disposable and can simply be discarded if the procedure fails.

## Self-learning and improvement

Beyond handling errors during synthesis, an ideal autonomous platform would learn and improve over time just as a chemist accrues knowledge and experience throughout their career. Arguably, these attributes of continual learning and the ability to respond to unforeseen outcomes are what would make a platform autonomous, rather than merely automated.

There are at least two factors that complicate this goal of life-long learning for data-driven platforms. First, the volume of data generated by a single platform will be overshadowed by the historical reactions tabulated in reaction databases; for new data to influence predictions, it may need to be treated separately by the algorithms (e.g., as a fine-tuning set) rather than integrated with a broader knowledge base. Second, the type of generated data will be qualitatively different from existing databases: it has the potential to be far richer in terms of procedural details and analytical chemistry, but will likely be unable to match the substrate diversity of published reactions given a practically sized chemical inventory. How to leverage this multi-modality is a new challenge in algorithm design.

## Outlook

Many elements of an autonomous platform for data-driven organic synthesis exist, yet we will continue to be stuck in the proof-of-concept phase unless several shortcomings are resolved. The level of precision required to execute synthetic pathways is not matched by the planning algorithms, and key challenges such as purification design remain almost entirely unaddressed. Data availability is a particular impediment, although nascent efforts like the Open Reaction Database^17^ may address this in time. Transitioning from “automation” to “autonomy” implies a certain degree of adaptiveness that is difficult to achieve with the limited analytical capabilities of many platforms. While already useful in isolation, these platforms will be particularly enabling when integrated with molecular design algorithms for *function*-oriented synthesis. In this setting, one can reconsider the role these platforms are meant to play: the ability to achieve any target molecular function may be more important than the ability to achieve any target molecular structure, which could make certain platform limitations (e.g., in terms of scope of reaction types) perfectly acceptable.
